# Cytosolic UDP-Gal biosynthetic machinery is required for dimerization of SLC35A2 in the Golgi membrane and its interaction with B4GalT1

**DOI:** 10.3389/fmolb.2025.1563384

**Published:** 2025-03-31

**Authors:** Wojciech Wiertelak, Artem Pavlovskyi, Mariusz Olczak, Dorota Maszczak-Seneczko

**Affiliations:** ^1^ Department of Biochemistry, Faculty of Biotechnology, University of Wroclaw, Wroclaw, Poland; ^2^ Novo Nordisk Foundation Center for Basic Metabolic Research, University of Copenhagen, Copenhagen, Denmark

**Keywords:** UDP-galactose, Golgi apparatus, glycosylation, SLC35A2, B4GALT1, protein-protein interactions

## Abstract

Glycosylation is a vital post-translational modification involving the addition of sugars to proteins and lipids, facilitated by glycosyltransferases and dependent on nucleotide sugar donors like UDP-galactose (UDP-Gal). This study examines how disruptions in UDP-Gal synthesis affect protein-protein interactions critical for glycosylation. Using CRISPR/Cas9, we generated HEK293T cell lines lacking key enzymes of the Leloir pathway: UDP-galactose 4′-epimerase (GALE), galactose-1-phosphate uridylyltransferase (GALT), or both. The knockout of GALE led to a significant reduction in intracellular UDP-Gal levels and altered N-glycan profiles, indicating impaired galactosylation. Through the NanoBiT assay, we observed that knocking out GALE alone or both GALE and GALT diminished the ability of the UDP-Gal transporter SLC35A2 to form homomers and to interact with the beta-1,4-galactosyltransferase 1 (B4GALT1). These findings suggest that the nucleotide sugar availability and/or the presence of the corresponding enzymes in the cytoplasm influences the formation of protein complexes involved in glycosylation in the Golgi apparatus, potentially affecting the glycosylation process itself. Our study highlights the dynamic nature of the glycosylation machinery and suggests that the interactions between glycosylation proteins are responsive to changes in nucleotide sugar levels. This opens new avenues for understanding the mechanisms underlying glycosylation and for investigating congenital glycosylation disorders.

## 1 Introduction

Glycosylation is one of the most common post-translational modifications in most eukaryotic cells, affecting macromolecules such as glycoproteins, proteoglycans, and lipids. The synthesis and modification of the glycan components are carried out by glycosyltransferases with catalytic domains located within the lumen of the endoplasmic reticulum (ER) and Golgi apparatus (GA). The substrates in glycosylation reactions are activated sugar molecules, known as nucleotide sugars. One of them is UDP-galactose (UDP-Gal). In human cells, UDP-Gal is synthesized in the cytosol through the Leloir pathway that involves four enzymes: galactose mutarotase (GALM), which converts beta-D-galactose into alpha-D-galactose; galactokinase (GALK), which converts galactose into galactose-1-phosphate; galactose-1-phosphate uridylyltransferase (GALT), which transforms galactose-1-phosphate and UDP-glucose (UDP-Glc) into UDP-galactose (UDP-Gal) and glucose-1-phosphate; and UDP-galactose 4′-epimerase (GALE), which, in turn, epimerizes UDP-Glc to form UDP-Gal [1]. The schematic representation of the Leloir pathway is shown in Supplementary Figure S1.

The synthesized UDP-Gal must then be transported into the lumen of GA/ER, where it serves as a substrate for glycosylation reactions. The transport of nucleotide sugars into these organelles is facilitated by a specialized family of proteins classified into the SLC35 family. These are multi-transmembrane proteins that function as antiporters, transporting nucleotide sugars in exchange for molecules of respective mononucleotides (Parker and Simon, 2017). To date, the only protein from the SLC35 family for which substrate specificity towards UDP-Gal has been established is SLC35A2 (Miura et al., 1996; Maszczak-Seneczko et al., 2011a; Maszczak-Seneczko et al., 2011b; Wiertelak et al., 2023a).

Glycosyltransferases are typically type II membrane proteins, characterized by a short N-terminal cytoplasmic tail, a single transmembrane domain (TMD), a stem region, and a C-terminal catalytic domain that resides in the lumen of the GA/ER (Breton et al., 2001; Grabenhorst and Conradt, 1999). One of the glycosyltransferases involved in galactosylation is the beta-1,4-galactosyltransferase 1 (B4GalT1). It plays a crucial role in the formation of complex type N-glycans (Bydlinski et al., 2018).

Protein-protein interactions (PPIs) play a crucial role in numerous biological processes, such as the enzyme-driven addition of carbohydrate groups to macromolecules. In recent years, the significance of PPIs in glycosylation mechanisms has become increasingly recognized. Notably, several studies have highlighted the formation of heterologous complexes by glycosylation enzymes that catalyze sequential reactions within a specific pathway (Kellokumpu et al., 2016; Wiertelak et al., 2024). Nucleotide sugar transporters (NSTs) also undergo homo- and heteromerization (Maszczak-Seneczko et al., 2012; Puglielli et al., 1999; Puglielli and Hirschberg, 1999; Wiertelak et al., 2020a). Parker et al. suggested that lipid-mediated interactions between NST monomers play a significant role in the molecular mechanism of transport (Parker et al., 2019).

Many of such interactions also occur between NSTs and glycosyltransferases (Maszczak-Seneczko et al., 2015; Khoder-Agha et al., 2019). So far, little is known about the biological significance of these interactions. However, in 2020, our team demonstrated the proximity between SLC35A2 and B4GalT1 as well as between two molecules of SLC35A2 using the NanoBiT technique (Wiertelak et al., 2020b). Additionally, our team demonstrated the interaction of SLC35A2 with the galactosyltransferase C1GalT1, which is responsible for the synthesis of mucin-type O-glycans. In cells lacking a functional SLC35A2 transporter, a reduced level of C1GalT1 and partial delocalization of this glycosyltransferase were observed (Wiertelak et al., 2023b), suggesting that a direct interaction between the transporter delivering the substrate and the enzyme utilizing it may be beneficial for the glycosylation reaction. Additionally, in 2022, it was demonstrated that mutated and less active versions of the CMP-Sia transporter (SLC35A1) exhibit a reduced ability to interact with the sialyltransferase ST3Gal4 (Wiertelak et al., 2022). These pathogenic variants, which are known to cause congenital disorders of glycosylation, also lost their ability to dimerize in living cells (Szulc et al., 2020).

The next step in our research was to determine whether the availability of the substrate could influence the interactions between transporters and the interactions between transporters and transferases. To this end, we established stable cell lines with partially or completely disrupted UDP-Gal synthesis and then investigated the ability of the SLC35A2 protein to homodimerize and interact with the galactosyltransferase B4GALT1.

## 2 Materials and methods

### 2.1 Cell culture maintenance

Wild-type HEK293T cells (ATCC CRL-3216) were grown in Dulbecco’s Modified Eagle’s Medium (DMEM, Sigma-Aldrich, St. Louis, MO, United States) supplemented with 10% fetal bovine serum, 100 U/mL penicillin, and 100 μg/mL streptomycin under standard conditions (37°C, 5% CO_2_). The cells were purchased from ATCC (American Type Culture Collection, Manassas, VA, United States).

### 2.2 Plasmid construction

Expression plasmids encoding human B4GALT1 (NM_001497.4) and SLC35A2 (NM_005660.3) fused with large and small NanoBiT fragments were generated using conventional restriction/ligation techniques according to the manufacturer’s protocols of the respective systems. Template cDNA was synthesized on total RNA isolated from wild-type HEK293T cells using LunaScript® RT SuperMix Kit (NewEngland Biolabs, Ipswich, MA, United States). Plasmids used in this study are detailed in our previous publication (Wiertelak et al., 2020b).

### 2.3 Generation of knockout cell lines

Three cell lines were generated with the help of the CRISPR/Cas9 gene editing approach using guidelines provided by Dharmacon (Lafayette, CO, United States). The first was lacking the *GALE* gene, whereas the second was lacking the *GALT* gene. The sgRNA sequences targeting *GALT* (SQ-010327-01–0002) and *GALE* (SQ-009904-01–0002) genes are listed in Supplementary Table S1. Then, based on the cell line with an inactive *GALT* gene, a double knockout cell line deficient in both GALT and GALE proteins was generated. HEK293T wild-type cells were co-transfected with a set of sgRNAs targeting three different regions of each gene and a Cas9-encoding plasmid according to the manufacturer’s instructions. In parallel, HEK293T wild-type cells were co-transfected with a set of non-targeting crRNAs (lot. U-007501–01-05), tracrRNA complexes (lot.U-002005–50) and a Cas9-encoding plasmid in order to generate control cells. The enrichment for gene-edited cells was done by growing transfected cells in complete DMEM medium, supplemented with puromycin (1 μg/mL) for 3 days. Single cell clones deficient in GALE, GALT and both GALE and GALT proteins were isolated and primarily screened via labeling of the whole cell protein with a biotinylated RCA I (*Ricinus communis agglutinin I*) conjugated with streptavidin-HRP (Vector Laboratories, Newark, CA, United States) followed by the analysis using dot-blotting. Gene editing in selected clones was further confirmed using RT-PCR and PCR performed on total cellular RNA and genomic DNA as templates, respectively. Total RNA was isolated using the EXTRACTME total RNA kit (Blirt). It was then reverse transcribed into cDNA using LunaScript® RT SuperMix (New England BioLabs). The resulting cDNA served as the template for PCR reactions performed with Q5® Hot Start High-Fidelity 2× Master Mix (New England BioLabs). Genomic DNA analysis was carried out using Platinum™ Direct PCR Universal Master Mix (Thermo Fisher Scientific), which enabled the use of whole cells as the starting material. All PCR and RT-PCR reactions were conducted according to the manufacturers’ protocols. The primers used are listed in Supplementary Table S1 and were synthesized by Microsynth AG (Balgach, Switzerland). Selected clones were finally confirmed using western blotting. Cells transfected with non-targeting sgRNAs and subjected to puromycin selection were used as control cells in all experiments and are further referred to as WT (these cells were not subjected to isolation of single cell clones).

### 2.4 Western blotting

Cells were lysed using CelLytic™ MT Cell Lysis Reagent (Sigma-Aldrich) supplemented with a protease inhibitor cocktail (Bimake) according to the manufacturer’s recommendations and protein concentration was estimated using a modified Bradford assay. Cell lysates were separated in SDS-PAGE using 12% polyacrylamide gels and proteins were electrotransferred onto nitrocellulose membranes. The membranes were blocked in 5% milk powder in PBS-T (1X PBS with 0.1% Tween-20) for 1 h at room temperature, then incubated overnight with the primary antibodies against GALT (lot. 17035-1-AP; Proteintech, Rosemont, IL, United States) and GALE (lot. sc-390407; Santa Cruz Biotechnology, Dallas, TX, United States) in dilution 1:1000, followed by the detection with secondary antibodies conjugated with HRP, respectively anti-rabbit (lot. A0545; Sigma-Aldrich) and anti-mouse (lot. SA00001-1; Proteintech) in dilution 1:7500. Signal was detected with chemiluminescence reagent (SuperSignal™ West Pico PLUS Chemiluminescent Substrate, Thermo Scientific) and documented using ChemiDoc MP Imaging System (Bio-Rad).

### 2.5 Quantitation of intracellular nucleotide sugars

Nucleotide sugars were extracted from collected and frozen cells following a previously published protocol (Räbinä et al., 2001) (the cells were counted before collecting and freezing). The enriched pools of nucleotide sugars were separated using ion-pairing reverse-phase HPLC, as previously described (Nakajima et al., 2010), with some modifications. The column was replaced with an Inertsil (GL Sciences, Shinjuku-ku, Tokyo, Japan) ODS-4, 250 × 3 mm, with a 3 μm particle size, and the flow rate was set to 0.3 mL/min. Buffer A (100 mM potassium phosphate, pH 6.4, with 8 mM tetrabutylammonium hydrogen sulfate) was used for equilibration, and buffer B (a mixture of 70% Buffer A and 30% acetonitrile) served as the eluent. The separations were carried out using the following gradient: 0% for 20 min, from 0% to 72% over 25 min, from 72% to 77% over 10 min, and finally equilibrating at 0% Buffer A for 25 min. Nucleotide sugar samples were dissolved in Milli-Q water, and sample volumes of 5 μL or less were injected to initiate the separation. The column was connected to a Nexera Shimadzu HPLC system, with the detection performed at 254 nm using an SPD-M30A Diode Array Detector equipped with a high-sensitivity quartz cell. The concentration of nucleotide sugars was calculated using LabSolutions Software (Shimadzu, Kyoto, Kyoto, Japan) using the high-purity nucleotide sugar standards (Promega, Madison, WI, United States). The chromatogram showing separation of nucleotide sugar standards (UDP-Gal and UDP-Glc) is shown in Supplementary Figure S2A. The absolute quantification of nucleotide sugars was achieved by comparing detected signals to externally added reference compounds. Next, by referring to the starting number of cells and the estimated volume per cell (3.5 pL), intracellular concentrations of nucleotide sugars were estimated.

### 2.6 N-glycans purification, digestion and separation

Cells were collected and lysed. The protein concentration of each sample was adjusted to 4 mg/mL. The resulting lysates were treated with acetone (1:1) to concentrate the proteins. The precipitated proteins were then solubilized in glycoprotein denaturation buffer (*N*-glycosidase F deglycosylation enzyme pack, New England Biolabs) and subjected to enzymatic deglycosylation using 500 units of *N*-glycosidase F (New England Biolabs) for 18 h at 37°C.

The released glycans were purified, labeled with 2-aminobenzamide (2-AB) and digested with α1-2,3,6 Mannosidase to reduce the background derived from high-mannose structures. Briefly, the samples were treated with 4 units of α1-2,3,6 Mannosidase (New England Biolabs) in GlycoBuffer 4 (New England Biolabs) supplemented with 2 mm ZnCl_2_ for 24 h at 37°C. Selected samples were digested with 8 units of beta 1-4 galactosidase (New England Biolabs) and/or 20 units of alpha2-3,6,8,9 neuraminidase A (New England Biolabs) in 10 µL of 1x Glycobuffer 1 (New England Biolabs) at 37°C for 16 h. Digested glycans were separated on TSK-Amide-80 150 × 4.6 mm, three µm column that was standardized with partially digested, 2-AB-labeled dextran. The column was connected to Nexera HPLC System (Shimadzu), equipped with RF-20A XS fluorescence detector set at 330/420 nm (excitation/emission) and stored and analysed using LabSolution software (Shimadzu). The obtained fluorescence values were normalized using min–max normalization and presented in graphs as fluorescence over time.

### 2.7 NanoBiT

This technique is based on the reconstitution of the NanoLuc enzyme from its two fragments, which are fused to the proteins of interest. When the proteins come into contact with each other, the enzyme is reconstituted, leading to a detectable bioluminescent signal. One protein in the pair is tagged with the large subunit, while the other is tagged with the small subunit. All possible combinations are tested, and the generated relative luminescence is compared to the relative luminescence of the negative control, which consists of one of proteins of interest tagged with the small NanoBiT fragment and a recombinant HaloTag protein fused with the large fragment. When the luminescence value exceeds a threshold of a 10-fold increase, the signal is considered indicative of an interaction, according to the manufacturer’s instructions.

Wild-type HEK293T cells (2 × 10^4^) were seeded in a complete growth medium into a 96-well plate with white polystyrene wells and a flat, transparent bottom (Greiner Bio-One, Kremsmünster, Austria) and cultured under standard conditions (37°C, 5% CO_2_). 20–24 h after seeding, the cells were transfected with plasmids mixed in equal amounts (25 ng of each plasmid per well) using Fugene HD transfection reagent (Promega, Madison, WI, United States) at a ratio of 3 μL per 1 μg DNA. Luminescence was measured 20–24 h post-transfection using the Glomax Discover Microplate Reader (Promega, Madison, WI, United States). Immediately before the measurement, the medium was replaced with the serum-free OPTI-MEM medium (Life Technologies, Carlsbad, CA, United States) supplemented with 25 μL/well of Live Cell Reagent (Promega). Each experimental combination was tested alongside a corresponding negative control, which included one of the proteins of interest fused with the large NanoBiT fragment (LgBiT, Promega, Madison, WI, United States) and HaloTag fused with the small NanoBiT fragment (SmBiT, Promega, Madison, WI, United States).

### 2.8 Statistical analysis and graphs preparation

All statistical analyses and graphs were performed using GraphPad Prism 8 (GraphPad Software, CA, United States) and Python software (version 3.9) with the Statsmodels (version 0.14.4, https://www.statsmodels.org/), Matplotlib (version 3.7.0, https://matplotlib.org/) libraries. Schematic figures were prepared with images from (https://smart.servier.com/).

### 2.9 Dot-blotting analysis using lectins

Cell lysates (2 μL) were spotted onto the nitrocellulose membrane. After drying, the membranes were stained in Ponceau S solution to confirm equal spotting. The dye was then washed off with distilled water, and the membranes were blocked in Carbo-Free Blocking Solution (Vector Laboratories, Newark, CA, United States) for at least 1 h at room temperature. After blocking, the membranes were washed three times in TBST solution (Tris buffered saline with 0.1% Tween-20). Next, the membranes were incubated for 1–2 h at room temperature with lectins from *R. communis* (RCA) and *Maackia amurensis* (MALI and MALII, Vector Laboratories, Newark, CA, United States) diluted 1:1,000–1:1,500 in TBST. Next, the membranes were washed three times in TBST and incubated for 1 h at room temperature in a solution of streptavidin conjugated with HRP (Vector Laboratories, Newark, CA, United States), diluted 10,000 times in TBST. The membranes were rinsed again in TBST and the signal was developed using Western Lightning® ECL Pro reagent (Thermo Fisher Scientific). The membranes were documented using the ChemiDoc MP Imaging System (Bio-Rad).

### 2.10 Indirect immunofluorescence

Cells were immunostained following the protocol previously described in reference (Szulc et al., 2020). The primary antibodies used were anti-GM130 (cat. 610823, BD Transduction Laboratories, Franklin Lakes, NJ, United States), anti-B4GalT1 (cat. HPA010807, Sigma-Aldrich) and anti-SLC35A2 (cat. HPA036087, Sigma-Aldrich) at dilutions ranging from 1:100 to 1:200. Detection of these primary antibodies was carried out using secondary antibodies conjugated with Alexa Fluor Plus 555 anti-mouse IgG and Alexa Fluor Plus 488 anti-rabbit IgG (Thermo Fisher Scientific) at a dilution 1:1,000. Imaging was performed using a Stellaris 8 (Leica, Wetzlar, Germany) system equipped with a 63x NA 1.4 immersion objective.

## 3 Results

### 3.1 Knocking out the GALT and GALE genes has influence on intracellular UDP-Gal concentration and cell glycophenotype

**FIGURE 1:**
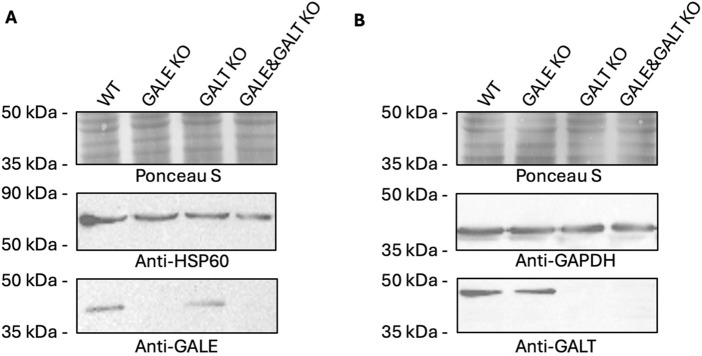
Western blotting analysis confirming the lack of functional proteins from the Leloir pathway. **(A)** Analysis using an anti-GALE antibody. **(B)** Analysis using an anti-GALT antibody. WT - wild type; GALE KO - GALE knockout; GALT KO - GALT knockout; GALE&GALT KO - double knockout of GALE and GALT. Ponceau S staining, anti-HSP60 and anti-GAPDH were used as loading controls.

One of the objectives of this study was to investigate the impact of deactivating genes encoding proteins from the Leloir pathway on the intracellular concentration of UDP-Gal. In the first step, we generated 3 cell lines: one lacking functional GALE protein (GALE KO), another lacking functional GALT protein (GALT KO), and a third line deficient in both proteins (GALE&GALT KO). In Figure 1, we presented clones that did not react with antibodies for the corresponding combinations in the western blotting technique. As can be seen, in the reaction with an antibody directed against the epitope present in the GALE protein, the signal from the band observed at approximately 38 kDa disappears in the GALE KO and GALE&GALT KO cell lines, in contrast to the wild type and GALT KO (Figure 1A; Supplementary Figure S3A). A similar situation can be observed for the anti-GALT antibody, where the signal from the band observed at approximately 43 kDa disappears in the GALT KO and GALE&GALT KO cell lines, while it remains present in the wild-type and GALE KO cells (Figure 1B; Supplementary Figure S3B).

**FIGURE 2:**
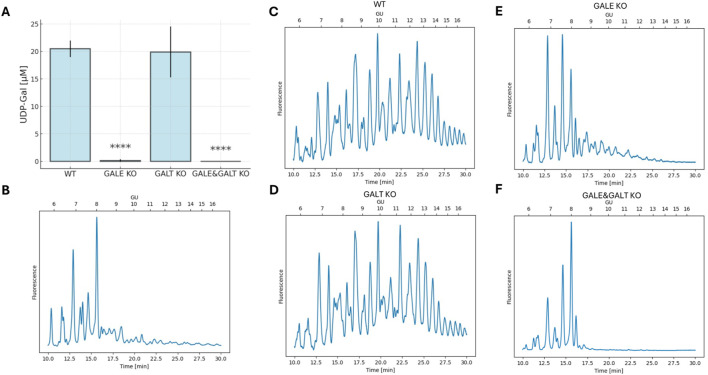
The impact of the absence of enzymes from the Leloir pathway on UDP-Gal concentrations and the glycophenotype. **(A)** Analysis of intracellular UDP-Gal concentration per cell. The graph shows the mean and standard deviation. Statistical significance was determined using one-way ANOVA followed by Tukey’s Honestly Significant Difference (HSD) test, based on three independent biological replicates. Asterisks indicate the level of statistical significance: p < 0.0001 (****) only in comparison to wild-type cell line. **(B)** HPLC separation of isolated, fluorescently labeled N-glycans from wild-type total cellular proteins digested with neuraminidase and galactosidase to remove galactose from the glycans. **(C–F)** HPLC separation of isolated, fluorescently labeled N-glycans from total cellular proteins. “Time [min]” refers to the retention time in minutes, and “GU” denotes glucose units. The cell lines included are wild type (WT), GALE knockout (GALE KO), GALT knockout (GALT KO), and GALE&GALT knockout (GALE&GALT KO). The presented result is representative of three independent experiments.

The next step was to examine the phenotype presented by these cell lines. To achieve this, nucleotide sugars were isolated from cell lysates, and their intracellular concentrations were measured. The fragments of HPLC chromatograms obtained for individual cell lines are shown in Supplementary Figure S3B-E. As can be seen in Figure 2, statistically significant differences were found only in cell lines that shared the common denominator of lacking the GALE protein. The cell lines lacking this functional enzyme exhibited an almost complete suppression of UDP-Gal synthesis. This contrasts with the knockout of the gene encoding GALT, in which the UDP-Gal levels did not change significantly. Although the difference between the GALE KO and GALE&GALT KO remains statistically insignificant, it is worth noting that the mean concentration of UDP-Gal in the GALE KO was slightly above the detection limit, whereas in the double knockout, none of the biological replicates exceeded it. The detection threshold was set at 0.2 μM.

Next, we decided to investigate how reduced intracellular concentrations of UDP-Gal, a substrate for galactosylation, affects the glycosylation of HEK293T cells (Figures 2C–F). To this end, we isolated N-glycans from the total protein pool in the cell and next, separated them using normal-phase chromatography. Less complex structures eluted first, while more complex structures, including those containing galactose, eluted later. The chromatograms for WT and GALT KO cells were very similar. The glycans were evenly distributed across the entire plot, indicating normal glycosylation in these cell types. However, in the case of GALE KO and GALE&GALT KO cells, we noted a significant shift in the peaks toward smaller structures. Moreover, we observed differences between the 2 cell lines lacking GALE; the one in which the GALT enzyme was present had more complex structures. To confirm that the disappearing peaks contain galactose, we compared the glycans with the same glycans treated with neuraminidase and galactosidase to remove galactose from the glycans. The resulting chromatograms were almost identical to the chromatogram obtained for the GALE&GALT KO (Figure 2B). To complement the HPLC results, we performed dot-blotting analyses using lectins specific for galactose (*R. communis* agglutinin, RCA; *M. amurensis* lectin I, MALI) and sialic acid (*M. amurensis* lectin II, MALII) (Supplementary Figure S3A). All these lectins displayed comparable reactivity with WT and GALT KO samples. In contrast, the reactivity of these lectins with lysates derived from GALE and GALE&GALT KO cell lines was dramatically decreased, confirming defective galactosylation.

### 3.2 The lack of UDP-Gal affects the interactions between proteins involved in galactosylation

**FIGURE 3:**
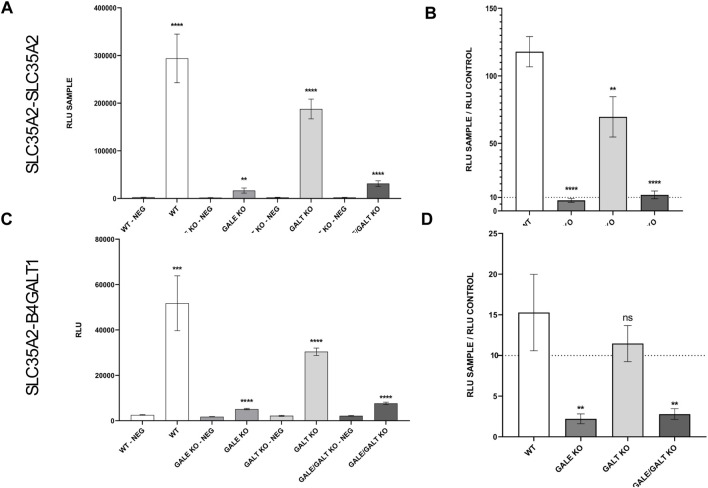
Results of NanoBiT analysis of homo- and heterologous interactions between SLC35A2 and B4GALT1 in HEK293T cells. **(A–D)** The cell lines included are wild type (WT), GALE knockout (GALE KO), GALT knockout (GALT KO), and GALE&GALT knockout (GALE&GALT KO). Negative control (NEG). RLU values obtained for the selected protein combinations and the corresponding negative controls. RLU, relative luminescence units. Data were analyzed using unpaired t-test and are presented as a mean ± standard deviation (SD) from three technical replicates **(A, C)**. Statistical significance was determined using one-way ANOVA followed by Tukey’s Honestly Significant Difference (HSD) test, based on three independent biological replicates **(B, D)**. **(A, C)** Asterisks indicate statistically important differences between tested combinations and corresponding negative controls. **(B, D)** Asterisks indicate statistically important differences between individual knockouts and wild-type cells. The significance level was set at p < 0.01 (**) and p < 0.0001 (****). **(B, D)** Ratios calculated by dividing a mean luminescence obtained for the tested combination by a mean luminescence obtained for the corresponding control. The dashed line marks a level of a 10-fold increase of signal over control which sets a threshold of significance suggested by the manufacturer.

The final step in the procedure was to examine how the reduction or complete absence of UDP-Gal affects the ability of SLC35A2 to homomerize, as well as the formation of heteromers between SLC35A2 and B4GALT1. For this purpose, the NanoBiT technique was employed. Dimerization of SLC35A2 and its interaction with B4GalT1 was described in more detail in our previous work, and the best performing combination based on our findings was selected (Wiertelak et al., 2020b). Figure 3A shows the behavior of the selected pair in the case of the SLC35A2-SLC35A2 interaction and its negative control across the different cell lines described in this study. In panel C (Figure 3C), we can see analogous pairs for the SLC35A2-B4GALT1 interaction. In panel B (Figure 3B), the relative ratio of the studied pair to its control for the SLC35A2-SLC35A2 interaction can be seen. In the GALT KO cell line, a significant decrease can be observed; however, it remains considerably above the threshold. The situation is different for the cell lines lacking functional GALE protein. Here, the signal is not only significantly different but also close to the threshold, suggesting a substantial defect in the formation of homomers by the UDP-Gal transporter. Similar results were obtained for the interaction between SLC35A2 and B4GALT1. In the GALE KO and GALE&GALT KO cell lines, a statistically significant difference and a decrease below the threshold were observed. In contrast, in the cell line lacking functional GALT, a slight decrease was observed, but it was not statistically significant (Figure 3D).

The defective formation of SLC35A2 homomers as well as SLC35A2-B4GalT1 heteromers could possibly result from their mislocalization as these complexes are expected to form in the Golgi apparatus. Therefore, we co-immunostained endogenous SLC35A2 and B4GalT1 with the Golgi marker GM130 in all analyzed cell lines (Supplementary Figure S5). The corresponding results demonstrate that the Golgi localization of SLC35A2 and B4GalT1 was not compromised in any of the knockout cell lines.

## 4 Discussion

In this study, we generated cell lines with defects in UDP-Gal biosynthesis to check whether the impairment of this biosynthesis will affect the interactions between the selected Golgi proteins involved in galactosylation of N-glycans. The effects of GALE and GALT deficiency were already studied by other research groups using yeast and mammalian cell culture models (Haskovic et al., 2020a; Ross et al., 2004; Mumma et al., 2008; Leslie et al., 1996; Broussard et al., 2020). Moreover, mutations in the human *GALT* gene are associated with a pathological condition termed galactosemia (Haskovic et al., 2020a), whereas mutations in the human *GALE* gene result in another disease called epimerase deficiency galactosemia (Oyanagi et al., 1981; Liu et al., 2013). In our study, the most significant reduction in intracellular UDP-Gal levels was observed in cell lines lacking functional GALE protein, specifically the lines referred to as GALE KO and GALE&GALT KO in this study. The intracellular concentration dropped almost below the detection limit in these cell lines. Since B4GALT1 galactosyltransferase, which is responsible for adding galactose residues to the synthesized N-glycans, is also a focus of this work, we investigated how the absence of UDP-galactose-4-epimerase affects the synthesis of these structures. The results seem to clearly suggest that the lack of this enzyme has a significant impact on the synthesis of galactose-containing N-glycans (Figure 2B). The results obtained in this study are consistent with data from the literature (Broussard et al., 2020).

It should be noted that GALT plays a significantly lesser role in the biosynthesis of UDP-Gal. Knockout of the gene that encodes GALT does not cause substantial changes in the concentration of this nucleotide sugar. This is in agreement with a previous study, in which knocking out the GALT-encoding gene in zebrafish did not cause any significant changes in nucleotide sugar levels, including UDP-Gal (Haskovic et al., 2020b). In cooperation with another enzyme from the Leloir pathway (GALK1), GALT converts galactose into UDP-Gal. Defects in this protein are more commonly associated with the disorder known as galactosemia rather than with glycosylation disorders (McCorvie et al., 2016). In 2005, Schulz et al. proposed that GALE acts as a “gatekeeper”, maintaining the balance of different nucleotide sugars, and that its absence, when cells are exposed to exogenous galactose, leads to the accumulation of high levels of UDP-Gal (Schulz et al., 2005). This suggests that GALT’s role is related to the utilization of excess galactose to a greater extent rather than its supply to N-glycans synthesis. It also appears that this may pertain to specific structures. A similar selectivity has been observed for GDP-fucose (Sosicka et al., 2022). Nonetheless, further studies on this topic are needed. In the final step, we investigated the ability of SLC35A2 to form homomers and associate with B4GALT1. Surprisingly, these interactions almost completely disappeared at reduced UDP-Gal concentrations. In 2019, Parker and colleagues conducted an experiment using a purified yeast GDP-mannose transporter, where they mixed active and inactive transporters in various ratios. They demonstrated that the functional form enabling the transport of nucleotide sugars across the membrane is a dimer (Parker et al., 2019). We hypothesize that, in the absence of the transported molecule, nucleotide sugar transporters remain in a ‘stand-by’ state in their monomeric forms, and only upon binding of a molecule to the transporter does the transporter form a dimer, allowing the molecule to be transferred into the lumen of the organelle. However, it cannot be excluded that this phenomenon is mediated by other proteins (either membrane or cytoplasmic) that on one hand are in contact with SLC35A2 or regulate its function by other means, and, on the other, somehow respond to the rates of UDP-Gal synthesis or the availability of GALE and GALT enzymes. Although the exact mechanism of the loss of SLC35A2 dimerization in the absence of the functional UDP-Gal biosynthetic machinery remains to be elucidated, this phenomenon appears remarkable and suggests a functional interplay between cytoplasmic nucleotide sugar biosynthetic machinery and transport of these compounds across the Golgi membrane, which clearly deserves further attention.

In the case of the B4GALT1-SLC35A2 interaction, the situation appears to be more complex. So far, the biological significance of this interaction has not been directly demonstrated. Additionally, B4GALT1 contains an N-glycosylation site (Yadav and Brew, 1990). Our team has shown, using another galactosyltransferase, B4GALT4, that improper glycosylation of the transferase can lead to protein delocalization, resulting in the loss of its interaction with SLC35A2 in the GA (Shauchuk et al., 2020). However, in this study we showed that the Golgi localization of endogenous B4GalT1 and SLC35A2 remains unaltered in all knockout cell lines, which precludes their mislocalization as a cause of defective complex formation. In one of our previous studies we have also demonstrated that inactive forms of the CMP-sialic acid transporter fail to associate with the sialyltransferase ST3Gal4. We believe that the lack of an active form of the transporter, which in the case of this study is due to the absence of UDP-Gal, leads to the absence of the interaction with the galactosyltransferase. In theory, a larger complex could form where the transported substrate might be delivered to the active site of B4GALT1 and then to the synthesized glycan within a single multiprotein assembly. Additionally, it is necessary to consider the possibility that the formation of complexes may also be influenced by other proteins, whose glycosylation may have been disrupted due to the lack of UDP-Gal synthesis. Moreover, it cannot be excluded that efficient interactions between SLC35A2 and B4GalT1 require not only the abundance of UDP-Gal but also the presence of an intact, functional and complete UDP-Gal biosynthetic machinery understood as a whole, i.e., GALK, GALT and GALE. In such a case, eliminating GALE alone could be sufficient to compromise the interactions between the Golgi galactosylation-related proteins even if UDP-Gal could be synthesized by GALK and GALT.

**FIGURE 4:**
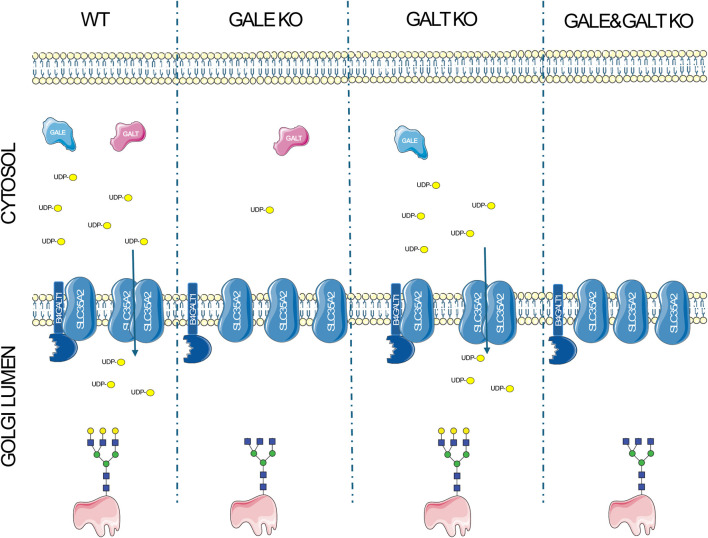
A schematic representation of the disruptions of protein complexes resulting from the impairment of the UDP-Gal biosynthesis pathway. In wild type cells, the synthesis of UDP-Gal proceeds without disruption in the presence of GALE and GALT enzymes, leading to the formation of homo- and heteromers between the UDP-Gal transporter (SLC35A2) and galactosyltransferase (B4GALT1), and resulting in the production of glycans containing galactose. The absence of GALE significantly reduces the intracellular concentration of UDP-Gal, decreases the number of complexes formed, and leads to the synthesis of glycans that lack galactose. In the case of GALT deficiency, there is a slight decrease in UDP-Gal concentration, which does not negatively affect the formation of complexes or glycans containing galactose in a significant way. The absence of both enzymes completely halts UDP-Gal synthesis, reduces the formation of complexes, and inhibits the synthesis of glycans with galactose in their structure.

In conclusion, we have demonstrated that there may be a potential link between the availability of nucleotide sugars and the interactions of proteins involved in glycosylation, which could impact the glycosylation process itself. Our data suggest that the complexes formed by proteins involved in glycosylation are not fixed assemblies but in turn undergo dynamic changes of their oligomeric state in response to the changes in the surrounding microenvironment (Figure 4). Our brief report opens new pathways for understanding the glycosylation process and for studying congenital glycosylation disorders. However, further studies are necessary to fully elucidate the relationship and significance of these interactions in the glycosylation process.

## Data Availability

The original contributions presented in the study are included in the article/Supplementary Material, further inquiries can be directed to the corresponding author.
