# Supplementary material for: Cytosolic UDP-Gal biosynthetic machinery is required for dimerization of SLC35A2 in the Golgi membrane and its interaction with B4GalT1
Source: Front Mol Biosci. 2025 Mar 31;12:1563384. doi: 10.3389/fmolb.2025.1563384 (PMC11994309; doi:10.3389/fmolb.2025.1563384)
Supplement: Supplementary file 1 [file Supplementaryfile1.pdf]

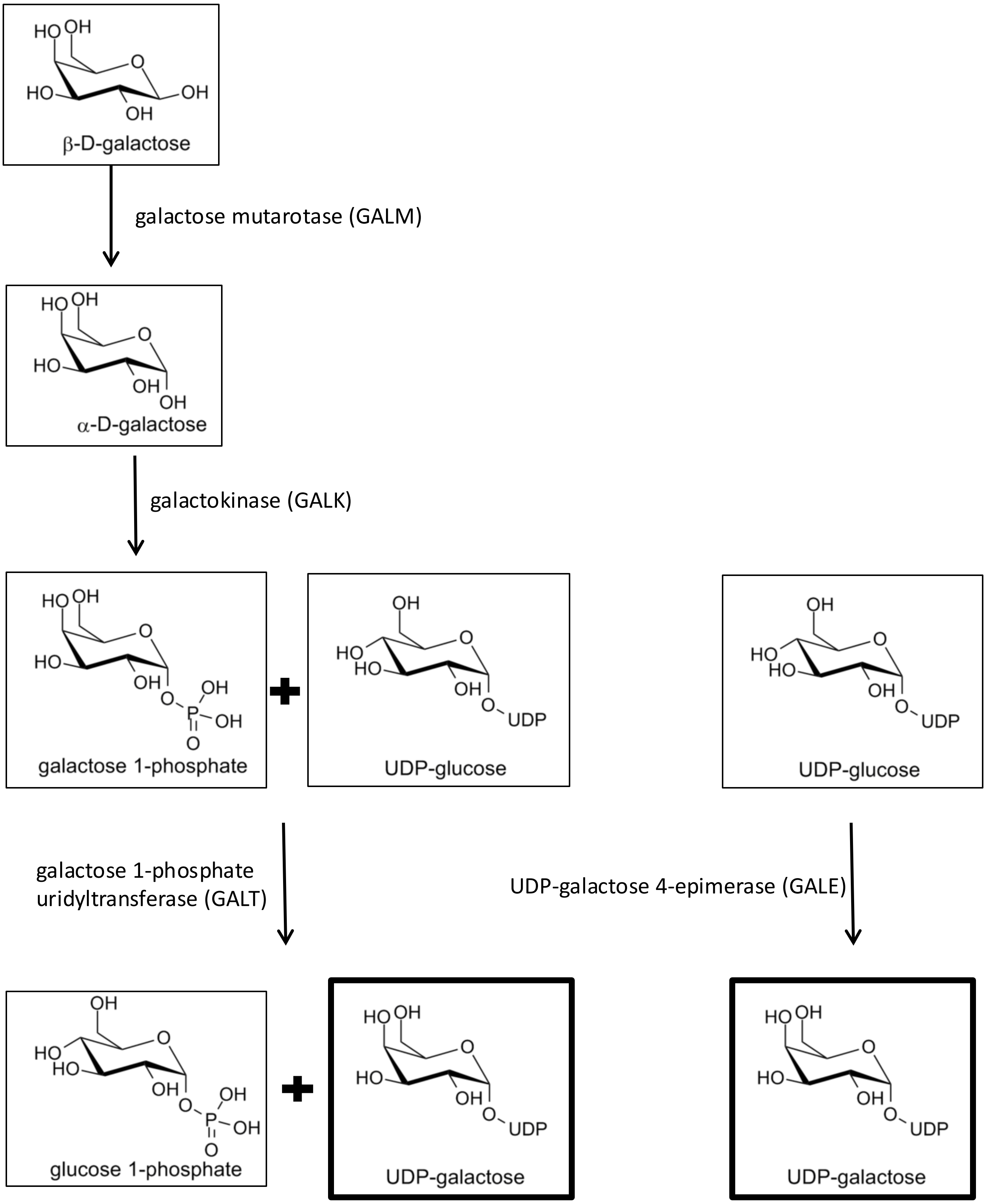

Supplementary Figure 1. Schematic representation of substrates, intermediates, final products and enzymes in the Leloir pathway of UDP-galactose synthesis.

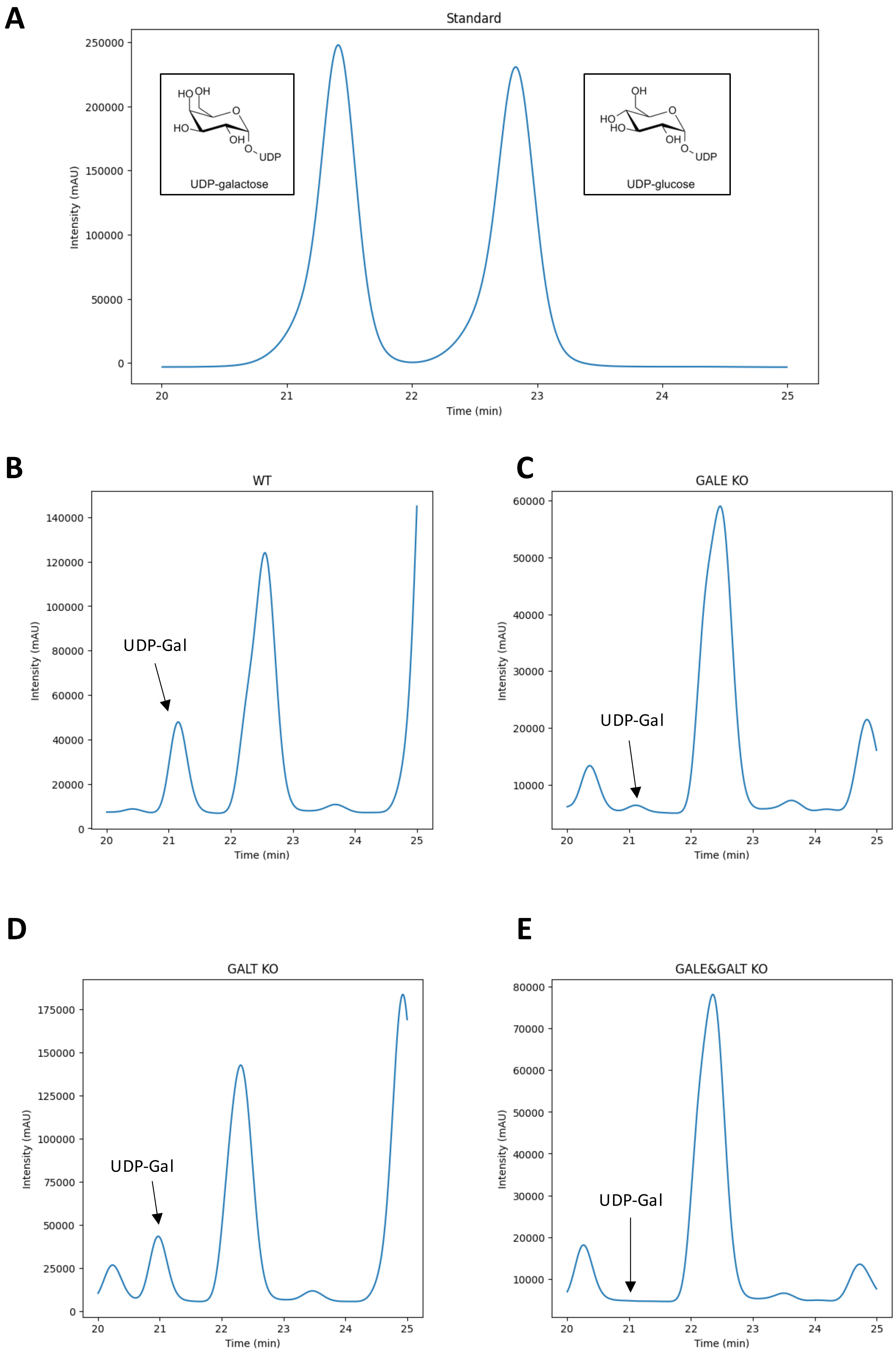

Supplementary Figure 2. HPLC separation of nucleotide sugars. (A) Separation of UDP-galactose and UDP-glucose standards. (B-E) Separation of nucleotide sugars isolated from different cell lines. WT - wild type; GALE KO - GALE knockout; GALT KO - GALT knockout; GALE&GALT KO - double knockout of GALE and GALT; UDP-Gal – UDP-Galactose.

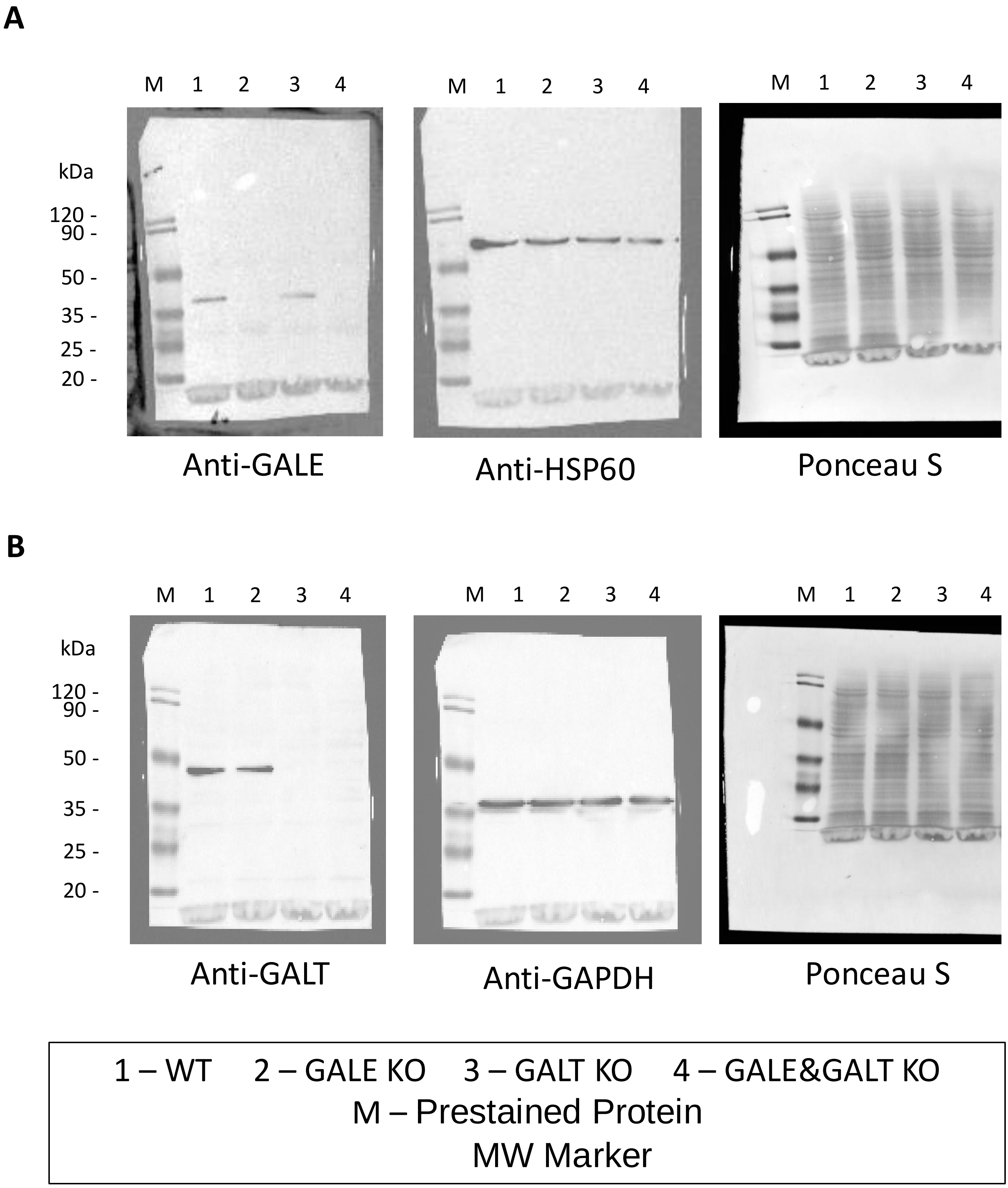

Supplementary Figure 3. Uncropped Western blots from Figure 1 confirming the absence of functional enzymes in the Leloir pathway. (A) Analysis using an anti-GALE antibody. (B) Analysis using an anti-GALT antibody. WT - wild type; GALE KO - GALE knockout; GALT KO - GALT knockout; GALE&GALT KO - double knockout of GALE and GALT. Ponceau staining, anti-HSP60 and anti-GAPDH were used as a loading controls.

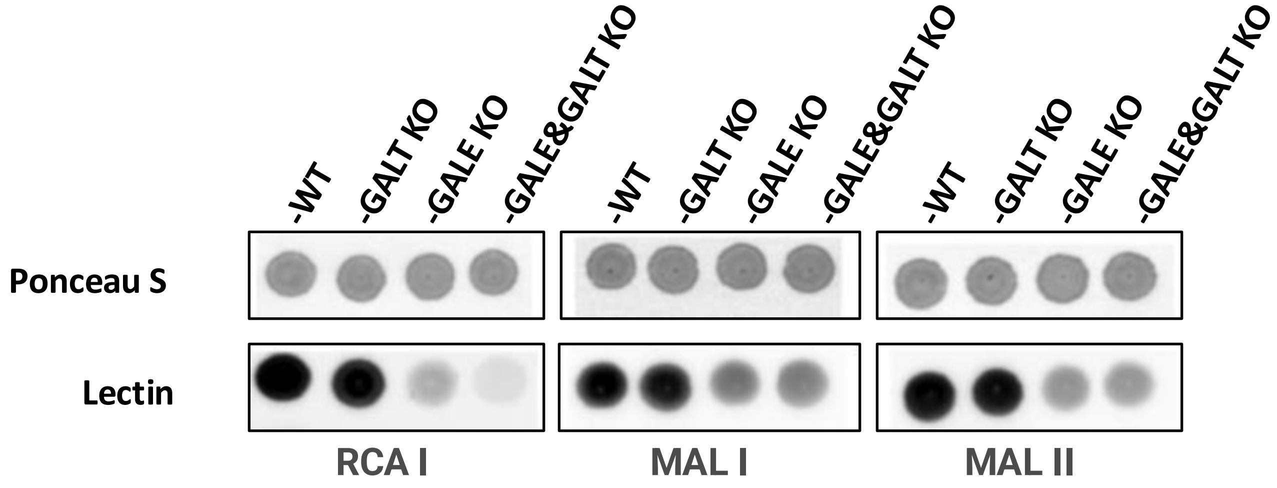

Supplementary Figure 4. Dot blot analyses showing a reduction in galactosylation in cells lacking functional enzymes of the Leloir pathway.WT - wild type; GALE KO - GALE knockout; GALT KO - GALT knockout; GALE&GALT KO - double knockout of GALE and GALT.

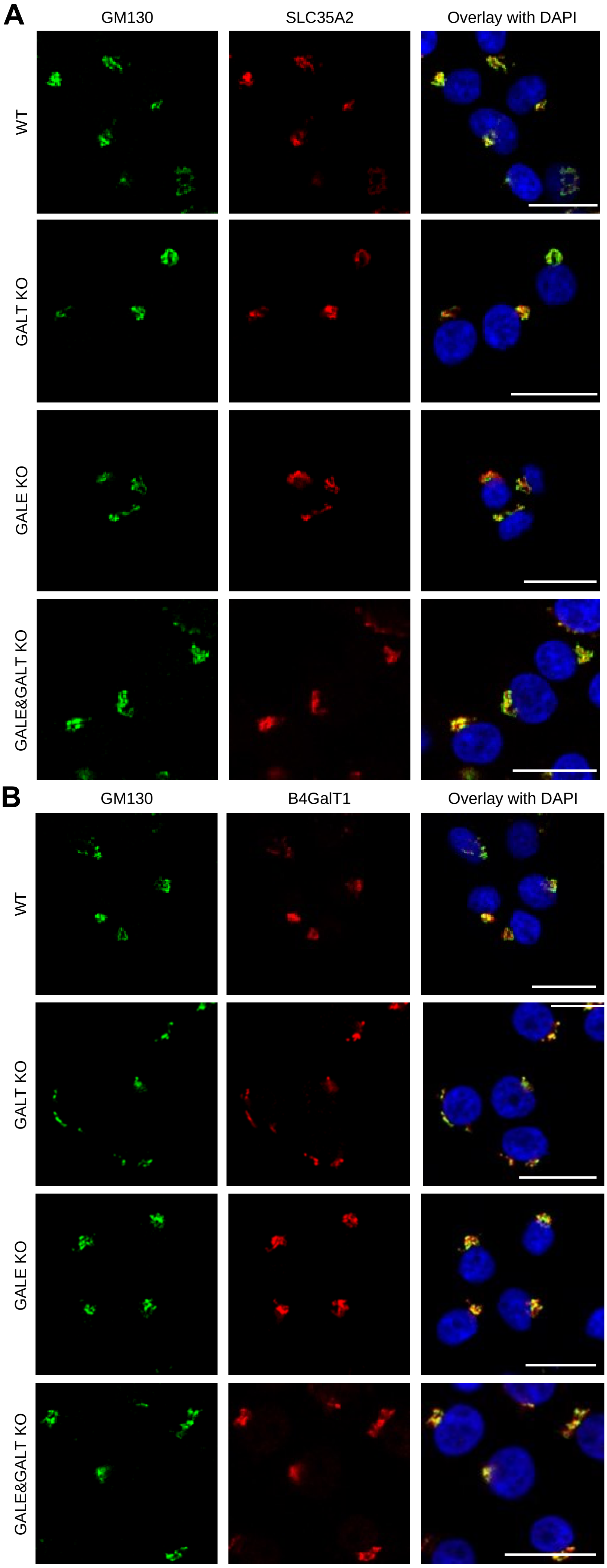

Supplementary Figure 5. Immunofluorescence stainings showing the colocalization of SLC35A2 (A) and B4GalT1 (B) with the Golgi marker protein (GM130) in the analyzed cell lines. WT - wild type; GALE KO - GALE knockout; GALT KO - GALT knockout; GALE&GALT KO - double knockout of GALE and GALT.

Template	Gene	Type	Sequence
cDNA	GALE	Forward primer	AAAAGAGCTCAGATGGCAGAGTGCT
		Reverse primer	AAAAGAATTCCCTCAGGCTTGCGTGCC
	GALT	Forward primer	AAAAGAATTCAATGTCGCGCAGTGGAA
		Reverse primer	AAAAGCTAGCTTAGGCGATGGTT GCTGT
gDNA	GALE	Forward primer	TCACTGATGCCATCTCTC
		Reverse primer	GCACAGGTCCCGGAT
		Forward primer	CTGAGACTCTGTATCCTG
		Reverse primer	CCCAGGGTTTTGCTT
	GALT	Forward primer	TTCTAGCCTATCCTTGTCG
		Reverse primer	AGAGGGGAAATCCATAGTTA
		Forward primer	CTTGATGACTTCCTA
		Reverse primer	CTCAAAGAGGTTGTCATAC

Supplementary Table 1. Primers used for screening clones in PCR experiments.
